# Protocol for promoting recovery optimization of walking activity in stroke (PROWALKS): a randomized controlled trial

**DOI:** 10.1186/s12883-018-1044-1

**Published:** 2018-04-12

**Authors:** Henry Wright, Tamara Wright, Ryan T. Pohlig, Scott E. Kasner, Jonathan Raser-Schramm, Darcy Reisman

**Affiliations:** 1 Department of Physical Therapy, University of Delaware, Newark, DE 19713 USA; 2 Biostatistics Core Facility, University of Delaware, Newark, DE 19713 USA; 3 Department of Neurology, University of Pennsylvania, Philadelphia, PA 19104 USA; 4 Christiana Care Health System, Newark, DE 19713 USA

**Keywords:** Stroke, Rehabilitation, Physical activity, Walking

## Abstract

**Background:**

Stroke survivors are more physically inactive than even the most sedentary older adults, and low activity is associated with increased risk of recurrent stroke, medical complications, and mortality. We hypothesize that the combination of a fast walking intervention that improves walking capacity, with a step activity monitoring program that facilitates translation of gains from the clinic to the “real-world”, would generate greater improvements in real world walking activity than with either intervention alone.

**Methods:**

Using a single-blind randomized controlled experimental design, 225 chronic (> 6 months) stroke survivors complete 12 weeks of fast walking training, a step activity monitoring program or a fast walking training + step activity monitoring program. Main eligibility criteria include: chronic ischemic or hemorrhagic stroke (> 6 months post), no evidence of cerebellar stroke, baseline walking speed between 0.3 m/s and 1.0 m/s, and baseline average steps / day < 8000. The primary (steps per day), secondary (self-selected and fastest walking speed, walking endurance, oxygen consumption) and exploratory (vascular events, blood lipids, glucose, blood pressure) outcomes are assessed prior to initiating treatment, after the last treatment and at a 6 and 12-month follow-up. Moderation of the changes in outcomes by baseline characteristics are evaluated to determine *for whom* the interventions are effective.

**Discussion:**

Following completion of this study, we will not only understand the efficacy of the interventions and the individuals for which they are effective, we will have the necessary information to design a study investigating the secondary prevention benefits of improved physical activity post-stroke. This study is, therefore, an important step in the development of both rehabilitative and secondary prevention guidelines for persons with stroke.

**Trial registration:**

ClinicalTrials.gov Identifier: NCT02835313.

First Posted: July 18, 2016.

## Background

Approximately 6.8 million adult Americans are living with stroke [1], which is a leading cause of serious, long-term disability in the US [1]. As a group, stroke survivors are even more physically inactive than the most sedentary older adults [2–4]. Lack of physical activity after stroke has serious consequences, including increased risk of a second stroke [5], developing other diseases and mortality [2, 3, 6].

Despite the severe consequences of inactivity, little attention has been paid to whether activity is influenced by rehabilitation interventions for chronic stroke survivors [7–11]. The few intervention studies that have examined walking activity generally found no improvement in activity [7, 9, 11], despite significant improvements in measures of walking capacity (e.g.-speed and endurance). This suggests that simply improving walking capacity is not sufficient for improving daily physical activity after stroke. One intervention, fast treadmill training, stands out for its ability to improve daily walking activity and capacity in chronic stroke survivors [8, 12, 13]. Our extensive study of fast walking training in chronic stroke suggests that improvements may occur through improved biomechanics and energy cost that impact walking speed and endurance [12, 14–17]. However, even with the gains with fast walking training, total daily walking activity (~ 4500 steps/day) [8] was far below recommended levels [18].

A recent meta-analysis found that step activity monitoring is an extremely effective stimulus for increasing daily walking activity in many patient populations [19]. The key ingredients of these programs are: 1) monitoring step activity with a pedometer or similar device, 2) setting a daily step activity goal and 3) identifying barriers to activity and strategies to overcome them [20–23]. Recent evidence suggests that a step activity monitoring program also improves walking activity in persons with chronic stroke [24]. Even with the gains, however, subject’s activity was still well below recommended amounts [24].

Together, these results led us to hypothesize that the combination of a fast walking intervention that improves walking capacity, with a step activity monitoring program that facilitates translation of gains from the clinic to the “real-world”, would generate greater improvements in real world walking activity than either intervention alone. Data provides support for this hypothesis; however, it suggests that the greater efficacy of combining the 2 interventions depends on a subject’s initial walking activity [25]. This is similar to previous rehabilitation trials in persons post-stroke that have also demonstrated variable effects of interventions, with some individuals showing improvements and others none [14, 26, 27]. Thus, *we do not expect that one intervention will be superior to the others for all subjects*, but rather that the combined intervention will be superior for those with low levels of baseline walking activity, speed and endurance. This type of specific hypotheses acknowledges that it is unlikely that one intervention is best for all after stroke.

The specific objective of this research study is to test *whether and for whom* combining fast walking training with a step activity monitoring program (FAST+SAM) is superior in improving real-world walking activity compared to fast walking training alone (FAST) or a step activity monitoring alone (SAM) in those with chronic stroke. Further, we aimed to explore the effect of these interventions on cerebrovascular and cardiovascular events, and risk factors in chronic stroke survivors.

## Methods/design

### Type of design

**Fig. 1:**
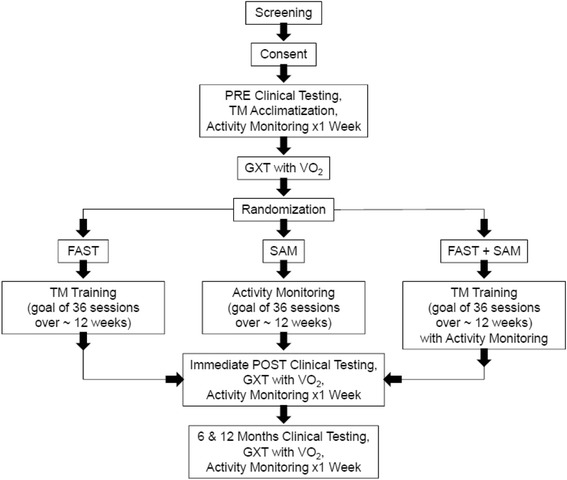
Study Schedule and Subject Flow Diagram (TM = Treadmill, GXT = Graded eXercise Test, VO2 = Ventilated Oxygen)

This is a single blind randomized controlled trial in which subjects are randomly assigned to one of three groups to test *whether and for whom* combining fast walking training with a step activity monitoring program (FAST+SAM) is superior in improving real-world walking activity compared to fast walking training alone (FAST) or a step activity monitoring and feedback program alone (SAM) in those with chronic stroke. Subjects undergo PRE testing prior to randomization and then complete the intervention. Subjects then undergo testing at ~ 12 weeks POST baseline and then return for FOLLOW-UP testing at 6 and 12 months post-baseline. The evaluator is blinded to group assignment at each time point. The primary outcome measure is steps per day and the secondary outcome measures include self-selected and fastest walking speed, walking endurance, and oxygen consumption. Exploratory outcomes include vascular events, blood lipids, glucose, and blood pressure. The sequence from screening, enrollment to randomization is represented in Fig. 1.

### Study enrollment

We plan to enroll a total of 258 individuals with chronic stroke during the proposed 5-year project. Eighty-six subjects will be recruited at each of our 3 sites: University of Delaware (UD), University of Pennsylvania (UPenn) and Christiana Care Health System (CCHS). Subjects are recruited from a variety of sources including existing clinical databases, local support groups, local physical therapy clinics, and advertisements.

### Informed consent

Subjects who are interested in participating sign informed consent prior to any study interventions.

### Screening process

Regardless of recruitment method, a member of the research team describes the purpose and general procedures of the study via an initial phone call or email. A Study Coordinator describes what to expect and the potential risks and benefits of participation. Potential subjects are also informed that joining the study is completely voluntary and that he/she is free to reconsider or stop participation at any time. If the potential subject expresses interest in agreeing to join, a series of screening questions to determine if the person might be eligible takes place.

### Inclusion & exclusion criteria

Inclusion criteria for participation in this study include: 1) Age 21–85, 2) Chronic stroke (> 6 months post stroke), 3) Able to walk at self-selected speed without assistance from another person (assistive devices are allowed), 4) Self-selected walking speed greater than 0.3 m/s and less than 1.0 m/s, 5) Average steps/day < 8000, 6) Resting heart rate between 40 and 100 beats per minute, 5) Resting blood pressure between 90/60 to 170/90.

Exclusion criteria for participation in this study include: 1) Evidence of cerebellar stroke, 2) Other potentially disabling neurologic conditions in addition to stroke, 3) Lower limb Botulinum toxin injection < 4 months earlier, 4) Current participation in physical therapy, 5) Inability to walk outside the home prior to the stroke, 5) Coronary artery bypass graft, stent placement or myocardial infarction within past 3 months, 6) Musculoskeletal pain that limits activity, 7) Inability to communicate with investigators, 8) score > 1 on question 1b and > 0 on question 1c on the NIH Stroke Scale.

### Assessments

#### Evaluation phases

Subjects participate in 4 evaluation phases during the course of the study as follows: PRE intervention evaluation phase, POST ~ 12-week intervention phase, 6 month FOLLOW-UP (FU6), and 12 month FOLLOW-UP (FU12). The POST and FU evaluation phases follow the same procedures as the PRE Intervention Evaluation phase for clinical testing, activity monitoring and GXT/V02 testing. At FU6 and FU12, subjects are also asked specifically if they had: recurrent stroke, myocardial infarction, unstable angina requiring hospitalization or peripheral artery event and the date of that event.

#### Clinical evaluation measures

At the start of the clinical evaluation the following measures/information are collected: a) Resting Blood Pressure (BP) taken according to American Heart Association guidelines [28], b) Resting Heart Rate (RHR) heart rate is averaged over 1 min as determined using a Polar Heart Rate Monitor, c) Body Mass Index (BMI) is calculated as mass divided by height squared. Height and weight are measured on the same medical scale at each evaluation, d) Medications List - Subjects are asked to provide the researcher with all the names of prescribed medications and any over the counter medications. Updates are made to the medication list at POST and FU testing if any changes have occurred, e) the Charlson Index [29] is a comorbidity scoring system that includes weighting factors on the basis of disease severity. Using the subject’s past medical history, the clinician assigns scores to each section and calculates the total score. Other past medical history, beyond that listed in the Charlson Index, is also noted during the clinical evaluation.

For the following measures, subjects utilize their preferred assistive device that they typically use at home or in the community and a gait belt or harness is used by a Research Physical Therapist for safety.

- **Self-Selected Speed (SSWS) and Fast Walking Speed (FWS)**: Walking speeds are assessed via the 10 m walk test for both SSWS and FWS. Subjects start 2 m back from the 6 m start line and continue for 2 m after the 6 m end line. Speed is averaged over 3 trials and measured in meters/s. This assessment has been used as a measure of short-distance walking speed in people post-stroke [30].
- **Six Minute Walk Test (6MWT)**: This is a test of endurance in which subjects are asked to walk continuously as fast as they can for six minutes so that when they are finished they walked as far as they could. Subjects walk around a rectangular path (4 turns required & approximately 42 m in total distance) until the 6 min time has elapsed. Subjects can stop and rest or sit and rest at any time. As talking can affect performance, talking only occurs if they have any questions or they want to tell the researcher how they are feeling. If subject has requested a rest break, the researcher lets them know they can take as much time as necessary. The researcher continues to provide subjects with a time elapsed statement at 1 min intervals. Total distance walked is recorded. This test has been used as an estimate of endurance in people post-stroke [31].

#### Questionnaires

Prior to the physical clinical testing, questionnaires are completed with subjects in a designated quiet area. Subjects complete the following:

- **Activities Balance Confidence Scale (ABC)**: is a 16-item questionnaire that is a measure of balance self-efficacy. Item scores are averaged to determine an overall balance confidence score ranging from 0% to 100%. The ABC Scale has demonstrated high test-retest reliability, excellent internal consistency, and concurrent validity in individuals more than 1 year post stroke [32].
- **Patient Health Questionnaire**: is 9-item self-administered depression screening and diagnostic tool increasingly used in primary care and other medical populations and has been shown to have high diagnostic accuracy for both major depression and any depression in persons post-stroke [33].
- **Montreal Cognitive Assessment**: is an 11-item cognitive screening tool covering orientation and attention, memory, language, visuospatial skills, executive function, verbal fluency and abstract thought. Instructions for each item are provided to subjects. Maximum score is 30 [34].

#### Treadmill acclimatization

Following completion of the baseline PRE clinical tests only, subjects are acclimatized to treadmill walking at their comfortable speed and fast speeds. This portion of the session is to familiarize the subject with treadmill walking prior to the GXT and to determine speeds that are used during that test. During the Acclimatization, subjects wear an overhead safety harness and heart rate is monitored throughout. The subjects are allowed to hold onto the handrails. The treadmill is ramped up to 0.2 mph below the overground SSWS and increased by 0.1 mph every 10–15 s until subject begins to struggle as evidenced by: toe catching or significant deleterious change in biomechanics, and/or drifting backward on treadmill and has difficulty correcting forward, and/or significant increase in use of handrail support, and/or heart rate exceeds 80% of max HR via Karvonen formula(35) or score of > 17 on the RPE scale [35].

#### Activity monitoring

At each evaluation phase, The Fitbit ™ is used to measure daily step activity. A testing Fitibit is placed in an ankle strap and put on the uninvolved ankle of the subject. Testing Fitbits are set up with a facility email account that is not known to the subject to prevent them from checking data and receiving feedback on their computer or smartphone. The display of the Fitbit is covered over so the subject cannot receive feedback from the device. The subject is instructed to wear the monitor during waking hours, except when bathing. Step activity data is collected for at least 7 days. Subjects are asked to perform only their regular walking activity. Subjects are educated on the wear and care of the unit as well as donning/doffing techniques through demonstration and a handout to take home. Subjects are contacted daily to remind them to wear the Fitbit. For those who are given a Fitbit device as part of the intervention, this is NOT the device that will be worn for testing at POST and FU phases. This is critical to prevent subjects from tracking their steps/day during the testing periods.

#### Graded exercise test with peak VO_2_

Prior to beginning the intervention, all subjects undergo a symptom limited GXT on a motorized treadmill. During the GXT, a 12-lead electrocardiogram (ECG) and breath by breath analysis of VO_2_ with a metabolic cart are recorded. Resting vital signs and ECG are obtained and then the subject walks on the treadmill at 85% of their fastest speed on the treadmill determined at the PRE treadmill acclimatization for 2 min. Subjects then advance to 2% incline for the next 2 min. Speed remains constant throughout the GXT and the incline is increased by 2% every minute thereafter to peak volitional exertion. Rate of Perceived Exertion [35] (RPE) and blood pressure are also monitored throughout. Test termination criteria include a subject’s request to stop, gait instability, or other stop criteria according to the guidelines of the American College of Sports Medicine (ACSM). The results of the test are reviewed by a cardiologist for abnormal responses that would prevent the subject from safe participation.

#### Blood testing

A fasting blood draw takes place at each evaluation phase to measure blood lipids, triglycerides and blood glucose.

#### Standardization of assessments

Standardization of data collection methods is achieved through a systematic training and competency assessment program for all physical therapists (PTs) blinded to the intervention. Performance of all outcome measures is reviewed and approved by the Primary Testing Research PT to ensure standardization. Continual training and feedback is provided to ensure sustained quality of outcome measures. Communication with the team regarding protocol modifications occurs through a group forum as well as in regular phone calls.

#### Randomization methods

Simple random sampling is used to randomize subjects into 1 of the 3 groups; FAST+SAM, FAST, or SAM. For allocation of the subjects, a computer-generated list of random numbers is used. The random allocation sequence is generated using https://www.randomizer.org. Three sets of numbers are generated, 1 set for each site, with 200 numbers per set. The number range used is 1–3 (1 = FAST+SAM, 2 = FAST, 3 = SAM). Three sets of sequentially numbered, opaque, sealed envelopes are generated (1 set for each site). Set #1 is for UD, set #2 is for CCHS and set #3 is for UPenn. Once a subject has successfully completed the GXT, the sealed envelope with their subject number is opened by the Study Coordinator who communicates the group assignment to the Research PT who will be completing the intervention portion of the study at the corresponding site. The researchers who are completing any of the evaluations are blinded to the subject’s group assignment.

#### Intervention

Subjects are randomly assigned to one of 3 groups: fast walking training (FAST), a step activity monitoring program (SAM) or a fast walking training + step activity monitoring program (FAST+SAM). The attendance goal for each group is 36 sessions at approximately 3× / week for 12 weeks.

#### Fast walking training

All subjects in the FAST and FAST+SAM groups complete a fast walking treadmill training program for 30 min, followed by approximately 10 min of overground walking activities each session. All treadmill walking is completed while subjects wear an overhead chest-harness system for safety; no body weight support is provided. Subjects walk for 30 min with the goal of walking at the fast training speed, one at which Target Heart Rate (THR) (calculated using the Karvonen formula) [36] is achieved: (THR) = (((max HR found on GXT) - Resting heart rate) × 70-80%) + Resting heart rate [36]. The fast training speed interval is reduced if: 1) the subject requests to walk slower, 2) the fast speed is no longer safe (e.g. increased toe scuffing or tripping), 3) the subject reports a rate of perceived exertion of ≥17 on the 6–20 Borg Rate of Perceived Exertion Scale [35], or 4) THR is exceeded. The treadmill speed is lowered to allow the heart rate to return to, (((max HR found on GXT) – Resting heart rate) × 50–60%) + Resting heart rate [36], or to a rate of perceived exertion ≤13. If the HR is not steadily decreasing and the recovery criteria are not achieved within approximately 1 min by walking slower, the treadmill is stopped and the subject takes a standing or seated rest break to achieve recovery. Once recovery is reached, the subject is transitioned to the fast training speed again.

Following treadmill walking, 10 min of over-ground walking activities are performed with the same THR and perceived exertion criteria as on the treadmill. Subjects are guarded and activities are progressed by the Research PT. The purpose of this portion of the session is for the subject to practice walking activities experienced during everyday activities (e.g. turning, backward stepping, walking while carrying objects) to gain both skill and confidence with these routine walking activities that are important in real-world walking.

During training, the RPE [35] is assessed every 2 min and heart rate is continuously monitored. Blood pressure is tested prior to the initiation of any activity. It is also checked at the Research PT’s discretion any time walking is stopped, including rest breaks, at the end of treadmill walking (prior to the start of over ground walking) and following over ground walking. For all sessions, the guidelines set forth by the ACSM for individuals in phase III or IV of cardiac rehabilitation are followed^1^. Based on these guidelines, we have developed session termination criteria. A session is terminated if any of the following occur: 1) Drop in Systolic blood pressure of ≥ 10 mmHg from baseline (resting for that day) despite increase in workload, 2) Hypertensive response with a systolic blood pressure > 240 mmHg and diastolic blood pressure > 110, 3) Presence of nervous system symptoms: ataxia, dizziness, or near syncope, 4) Any chest pain or angina symptoms, 5) Signs of poor perfusion cyanosis or pallor, or 6) Excessive fatigue, excessive shortness of breath, leg cramps, claudication. If any of the above occurs, the subject’s physician is contacted and informed of the subject’s response to the activity. Subjects can also terminate any session or training bout by stating their desire to stop.

#### Step activity monitoring

Subjects in the SAM and FAST+SAM groups participate in the step activity monitoring program and are provided with a Fitbit. If the device is lost or damaged, it is replaced to allow continued participation in the step activity monitoring program. Baseline step activity data for subjects in the FAST+SAM and SAM groups is used to categorize and assign step activity goals. It is important to note that goals are set not based on an absolute number for all subjects, but rather individually, based on their own baseline walking. Goals are advanced based on subject achievement of previous goals. Step activity data in the interim days between sessions is reviewed and used to assist subjects in understanding how much walking activity they performed during certain daily activities, like walking to the mailbox or walking laps around their home, and how that added to their total steps per day. Subjects are also advised of how their steps per day related to goal achievement; a discussion of individualized ideas to increase activity and of any barriers and how to overcome those barriers occurred. These discussions have been shown to be critical for the success of a step activity monitoring program.

Subjects in the FAST group are instructed not to begin the use of a step monitor during the study.

#### Standardization of intervention

The walking training and activity monitoring interventions are standardized so that implementation is consistent across clinical sites. Standardization assures that a common intervention is applied. Research PT’s demonstrate: 1) knowledge of the protocol; 2) decision making and progression of both the walking training and activity goal setting; 3) subject safety and monitoring; and 4) equipment use. Documentation is standardized across sites through the completion of walking training and activity monitoring session information in the REDCap PROWALKS Training Database.

A competency-based training program is used to train the Research PT’s at the 3 sites. First Research PT’s at each site are provided with the manual of operating procedures and instructed to review pertinent sections. The Primary Training Research PT provides training at each site, followed by a several-week period of hands on practice and problem-solving with weekly group (via phone) question and answer sessions. The Primary Training Research PT returns to each site to complete competency testing through observation of 2 pilot training sessions at each site. Competency is required in each of the 4 domains listed above. After individual Research PT’s achieve competency status, they are approved to admit subjects to the RCT. At least, bimonthly meetings of all clinical study personnel (in person and via phone or video-conferencing) address subject-specific issues related to intervention adherence.

Treatment fidelity is also maintained throughout the study through systematic review of treatment delivery. For the first subject at each site, treatment fidelity is assessed by the Primary Training Research PT after 2, 4, 8, and 10 weeks. For subsequent subjects, treatment fidelity is evaluated by the Study Coordinator and the Primary Training Research PT on a quarterly basis by assessing at least 1 subject from each group at each site with the goal of assessing treatment fidelity of each trainer within each group. Any intervention related issues are communicated to the site PI. Sessions may be video-taped to use for training and/or to further assess standardization compliance. A Treatment Fidelity webform is completed to track intervention standardization compliance. Turnover in Research PT’s across sites is anticipated and the Primary Training Research PT is responsible for training new staff and competency must be achieved as outlined above.

### Statistical analysis

#### Sample size determination

Our data from previous work suggests an improvement of ~ 1700 steps/day in those with low steps/day at baseline. Given this and other recent work [37], we would consider an average improvement of ≥1700 steps/day in the FAST+SAM group worthy of further study in future clinical trials. Power calculations indicate that a total of 225 participants are needed (75 in each of the 3 groups) in order to detect this differential increase based on baseline steps/day and intervention received with power greater than 0.90. These calculations assume equal group sizes, a moderate correlation among repeated measures of *r* = 0.50, and a standard deviation for steps/day of 2500 [8, 24].

#### General data management & analysis

A database containing subject numbers, demographic information and collected data is stored on a password-protected server only accessible to members of the research team. The Biostatistician running analyses is blinded to group assignment. In general, both an “intent to treat” and a “per protocol analysis” will be performed, if results between the two analyses differ the “intent to treat” results will be used for primary publication. To ensure that the randomization worked, potential covariates (e.g. age, time since stroke, cognition) will be compared between groups using t-tests and χ2 tests. If a covariate is significantly different between groups, this will be an indication that the groups are not balanced and the covariate will be included in the models. The alpha is set at 0.05 for primary analyses and .01 for secondary analyses.

To compare the efficacy of the FAST+SAM, FAST and SAM interventions for improving real-world walking activity in chronic stroke survivors and to determine for whom the interventions are most effective General Linear Mixed Models (GLMM) and moderated multiple regression models will be developed. All model assumptions will be tested (including linearity, normality, homoscedasticity, and multicollinearity as well as checking for outliers to be removed). Violations of the assumptions will be remedied using transformations or a Generalized Linear Model. Post-hoc tests in the GLMMS will use Bonferroni adjustment and for the regression models post-hoc probing of interactions will be done using simple slopes method. In addition to the moderating effects identified a priori (baseline steps/day, walking speed and endurance), other factors (balance self-efficacy, depression and co-morbidity burden) that might moderate the intervention will be tested as secondary analyses.

To determine the effect of the FAST+SAM, FAST and SAM interventions on cerebrovascular and cardiovascular events, and risk factors in chronic stroke survivors we will use a Cox Proportional Hazards Model for time to first cardiac event and a Generalized Estimating Equation, for the total number of cardiac events at the one year follow-up.

#### Adverse event monitoring & reporting

Study approval is in effect via the Institutional Review Boards (IRB) of the University of Delaware, the University of Pennsylvania and the Christiana Care Health System. All reporting guidelines from each institution are followed. No provisions are provided for ancillary or post-trial care.

Serious adverse events include death, life threatening event (stroke, myocardial infarction, fracture), inpatient hospitalization, a significant disability or incapacity that lasts 48 h and limits activities of daily living. Minor adverse events include fall without fracture, dyspnea, open sore, blister, cut, muscle soreness or pain that lasts more than 48 h, dizziness/fainting, diaphoresis. Serious adverse events are reviewed to determine whether they are unexpected.

Expected risks for this study are defined as: Risks due to physical activity including pain, fainting, dizziness, fatigue, nausea, shortness of breath, chest pain or angina, myocardial infarction, swelling, bruising, muscle/bone/joint soreness, joint damage, bone fracture, ligament/tendon/connective tissue damage, hospitalization, and death. Psychological risks include possible discomfort, frustration, and/or anxiety related to difficulty with the physical testing or completion of questionnaires. The risks of taking blood include pain and/or bruising where the blood is taken, redness and swelling of the vein and infection, and a rare risk of fainting.

We also consider whether an event is likely related to the study. This would be an event that reflects a realistic chance of a causal relationship between study intervention and the adverse event, as suggested when the event occurs within a short time frame after the intervention (24 h), follows a pattern consistent with study intervention, and improves when the study has stopped or reappears when the study is resumed.

#### Reporting

Monitoring and reporting adverse events, whether anticipated or unanticipated, is the responsibility of the Principal Investigator (PI). The reporting requirements of the UD, UPenn and CCHS Institutional Review Boards for serious and unexpected adverse events are followed. Adverse events are tracked by the Primary Training Research PT through a webform platform that directly populates an electronic spreadsheet. Should an adverse event occur, a webform is completed by the respective overseeing training Research PT. Once submitted, events are adjudicated by the study PI and if necessary the Data Safety Monitoring Board and / or the on-site Institutional Review Board is notified.

#### Data management & quality

Secure electronic database(s) using the REDCap platform have been developed for the study. The number of potential subjects screened, enrolled and disqualified is collected by the Study Coordinator through a webform platform that directly populates an electronic spreadsheet. Adherence and retention are tracked through the PROWALKS REDCap Training database and the PROWALKS REDCap Clinical database. Adverse events are tracked by the Primary Training Research PT through a webform platform that directly populates an electronic spreadsheet.

Data from each testing session is entered directly into the PROWALKS REDCap Clinical database. This database is designed with built in quality control checks (e.g., range checks, checking for missing data for required data points). In addition, each month the Primary Training Research PT examines frequency distributions of outcome variables to identify questionable data points.

Data from each training session is entered directly into the PROWALKS REDCap Training database. This database is designed with built in quality control checks (e.g., range checks, checking for missing data for required data points). In addition, each month the Study Coordinator reviews a random sampling of data from 6 sessions at each site according to the Treatment Fidelity checklist webform. Intervention adherence (e.g.-number of sessions completed out of a total possible 36) is tracked via the data in the PROWALKS REDCap Training database yearly by the Study Coordinator.

#### Study organization & management

The Data and Safety Monitoring Board (DSMB) acts in an advisory capacity to the Institutional Review Board at the University of Delaware to monitor subject safety, data quality and evaluate the progress of the study. In addition, the study undergoes regular internal audits and is subjected to external auditing through the UD IRB.

The DSMB for this study reviewed the research protocol, informed consent documents and plans for data safety and monitoring, will evaluate the progress of the trial, including periodic assessments of data quality and timeliness, recruitment, accrual and retention, subject risk versus benefit, performance of the trial sites, and other factors that can affect study safety and outcome, will make recommendations to the primary site Institutional Review Board and the Principal Investigator concerning continuation, termination or other modifications of the trial based on the observed progress and effects of the intervention under study, report to the funding agency on the safety and progress of the trial and assist the Institutional Review Board by commenting on any problems with study conduct, enrollment, sample size and/or data collection.

Meetings of the DSMB are held at least annually at the call of the Chairperson or other members of the DSMB. The first meeting of the DSMB occurred prior to the enrollment of the first subject to familiarize members with the research protocol, ensure the appropriateness of the informed consent documentation, and understand the proposed plan for safety and data monitoring of the study.

## Discussion

Post-stroke disability is strongly linked to inactivity. Stroke is the leading cause of disability in the United States [38]. Approximately 80% of the 5.5 million people living with stroke have some level of disability [39]. This disability is a consequence of, and a risk factor for, physical inactivity [40, 41]. The onset of disability makes it difficult for stroke survivors to engage in physical activity, causing them to remain sedentary and extremely inactive [40–42]. This inactivity can further functional loss and disability and thus, the vicious cycle continues [41]. In the chronic phase of stroke (> 6 months post-stroke), daily walking activity (generally 3500 steps/day or less) [2, 3, 43] is well below the activity level of even the most sedentary adults (< 5000 steps/day) [18]. The FAST+SAM intervention in this proposal is designed to interrupt this cycle through direct and indirect effects on physical inactivity after stroke.

Lack of physical activity has serious health consequences for chronic stroke survivors. Inactivity leads to an increased risk of a second stroke [5], an increased risk of developing other diseases such as diabetes and an increased risk of mortality [2, 3, 6]. The risk of myocardial infarction or vascular death in the first 5 years after stroke is 17.4% or just over 3% per year [44]. This means that those with stroke have equal long-term risk for a vascular event or MI as those with known coronary disease [3, 45]. In chronic stroke survivors, inactivity exacerbates the normal decline in aerobic fitness, putting most stroke survivor’s cardiovascular capacity below the level needed for activities of daily living [3, 46]. The potential consequences of inactivity are even more alarming considering that inactivity gets *worse* over the first year after stroke [5]. If the vicious cycle of disability ↔ inactivity can be interrupted through improvements in activity after stroke [41], then many negative sequela of stroke may also be avoided.

Traditional rehabilitation interventions have little effect on the physical activity of chronic stroke survivors. To our knowledge, only a handful of rehabilitation intervention studies have examined changes in physical activity as an outcome of the intervention [7–10, 47]. When activity is measured, the results indicate that current rehabilitation interventions have limited impact on the daily walking activity of chronic stroke survivors [7–10, 47]. These studies found either no improvement [7, 9, 47] or when an improvement was observed, the subjects still remained relatively sedentary, walking fewer steps per day than sedentary older adults and well below the recommended levels of walking activity [8, 10]. It is therefore critical that interventions are developed that can increase the physical activity of stroke survivors. It is likely that this may be effectively done by incorporating interventions known to improve physical activity in other populations into the rehabilitation of chronic stroke survivors. Step activity monitoring programs, which aim to increase real-world activity, increase walking in a variety of patient populations [19, 48]. Such programs are also effective for increasing real-world walking after stroke [24] and may be particularly effective in combination with walking rehabilitation interventions (such as FAST+SAM) after stroke because they promote translation of gains from the clinic to the “real-world”. Indeed studies suggest that adherence at both one and two years following the start of exercise is better with programs that can be completed in the home [49, 50].

Focus on an understanding of *for whom* the interventions are effective. Recent rehabilitation clinical trials in chronic stroke have demonstrated significant, but variable, effects of interventions with some individuals showing significant improvements and others no improvement [11, 26, 27]. Moreover, studies have demonstrated equivalent results between various, equally dosed therapies [51, 52]. Together, these results suggest that *some individuals do not respond to specific therapies* and there is growing recognition in the post-stroke rehabilitation research community that the focus of studies should not be simply on identifying which interventions are efficacious, but rather to identify *for whom* certain interventions are most efficacious [11, 53], acknowledging that one intervention is not likely best for all. The analyses in this study will therefore examine the interaction between potentially moderating baseline characteristics and the individual interventions. This will avoid erroneous conclusions that one intervention is not superior to another, when in fact; the intervention is superior for a sub-group of subjects. Data suggests that the greater efficacy of combining the 2 interventions depends on a subject’s initial walking activity; in particular, the combined intervention may be superior for those with low levels of baseline walking activity, speed and endurance [25]. Importantly, then, the results of this study will tell us not only whether one intervention is superior, but for whom that intervention is most effective.

Physical inactivity, disability and health are intimately related after stroke. Improvements in activity should therefore improve not only post-stroke disability, but also lead to a reduced risk of secondary health conditions. The expected positive results of this study will determine for whom particular interventions are most effective for improving real world walking activity after stroke. The next step in this research is to implement those interventions and examine the secondary prevention benefits.

## Abbreviations

6MWT: 6 Minute walk test
ABC: Activities balance confidence
ACSM: American College of Sports Medicine
BMI: Body mass index
BP: Blood pressure
CCHS: Christiana Care Health System
DSMB: Data and safety monitoring board
ECG: Electrocardiogram
FAST: Fast walking training
FAST+SAM: Fast walking training & step activity monitoring
FU12: 12 Month follow-up
FU6: 6 Month follow-up
GLMM: General linear mixed model
GXT: Graded eXercise Test
MACCE: Major Adverse Cardiac / Cerebrovascular Event
PI: Principal investigator
PT: Physical therapist
RHR: Resting heart rate
RPE: Rate of perceived exertion
SAM: Step activity monitoring
THR: Target heart rate
TM: Treadmill
UPenn: University of Pennsylvania
VO_2_: Oxygen consumption
